# Seroprevalencia y tasa de ataque clínica por chikungunya en Nicaragua, 2014-2015

**DOI:** 10.26633/RPSP.2017.59

**Published:** 2017-07-02

**Authors:** Ministerio del Poder Ciudadano para la Salud de Nicaragua

**Affiliations:** 1 Dirección General de Vigilancia para la Salud Ministerio del Poder Ciudadano para la Salud de Nicaragua Managua Nicaragua Dirección General de Vigilancia para la Salud, Ministerio del Poder Ciudadano para la Salud de Nicaragua, Managua, Nicaragua.

**Keywords:** Fiebre chikungunya, seroprevalencia, infecciones subclínicas, Arbovirus, Nicaragua, Chikungunya fever, seroepidemiologic studies, asymptomatic infections, Arbovirus, Nicaragua

## Abstract

**Objetivo.:**

Estimar la seroprevalencia, la tasa de ataque clínica y la proporción de infecciones subclínicas por chikungunya,

**Métodos.:**

Se realizó un estudio transversal en 39 sitios distribuidos en todo el territorio nacional de Nicaragua en octubre 2015. Se recopiló información demográfica y clínica a través de una encuesta personal. Se recolectaron muestras hemáticas para detectar la presencia de anticuerpos antivirus chikungunya utilizando el método de ELISA de inhibición desarrollado por el Centro Nacional de Diagnóstico y Referencia. Se utilizaron modelos lineales generalizados y modelos de multinivel de Poisson en el análisis de los resultados.

**Resultados.:**

Se enrolaron 11 722 participantes mayores de dos años de edad y se procesaron 11 280 muestras. En el nivel nacional, la seroprevalencia fue de 32,8% (IC95% [intervalo de confianza de 95%]: 31,9-33,6), con una tasa de ataque clínica de 26,5% (IC95%: 25,7-27,3) y una proporción de infecciones subclínicas de 19,1% (IC95%: 17,8-20,4). Se observó variabilidad en la seroprevalencia de los 39 sitios, y los que presentaron mayor índice de infestación por el vector mostraron una mayor seroprevalencia. A nivel individual, esta fue más elevada en los participantes mayores de 11 años.

**Conclusión.:**

*Este es el primer estudio sobre la seroprevalencia de chikungunya en América Latina continental desde su introducción, en el que se determinaron la prevalencia a nivel nacional, la tasa de ataque clínico y la proporción de infecciones subclínicas. El modelo utilizado, con una amplia participación comunitaria y el rol rector del Ministerio de Salud* *de Nicaragua, puede constituir un ejemplo para la realización de estudios similares en la región.*

## Abstract (en)

**Objective.:**

Estimate seroprevalence, clinical case rate, and proportion of subclinical infections from chikungunya.

**Methods.:**

A cross-sectional study was conducted in October 2015 at 39 sites distributed across Nicaragua. Demographic and clinical information was compiled through a personal survey. Blood samples were collected to detect chikungunya antibodies using the ELISA inhibition method developed by Nicaragua’s National Diagnostic and Reference Center. Results were analyzed using generalized linear models and multilevel Poisson models.

**Results.:**

A total of 11 722 participants aged >2 years were enrolled and 11 280 samples were processed. National seroprevalence was 32.8% (95% CI [95% confidence interval]: 31.9-33.6), with a clinical case rate of 26.5% (95% CI: 25.7-27.3) and a proportion of subclinical infections of 19.1% (95% CI: 17.8-20.4). Seroprevalence varied among the 39 sites and was greater at sites with higher vector infestation indices. Individually, seroprevalence was higher in participants aged >11 years.

**Conclusion.:**

Since its introduction, this is the first study on chikungunya seroprevalence in continental Latin America to determine national prevalence, clinical case rate, and proportion of subclinical infections. The study model, employing broad community participation and leadership by the Ministry of Health of Nicaragua, can be an example for conducting similar studies in the region.

El virus chikungunya (CHIKV) es un alfavirus artrogénico reemergente transmitido por los mosquitos *Aedes aegypti *y* Aedes albopictus* (1). La fiebre por virus chikungunya es una enfermedad caracterizada por la presencia de fiebre, artralgia intensa e inflamación de articulaciones y músculos, que puede causar incapacidad transitoria en los pacientes (2). Se han descrito formas atípicas del chikungunya, incluyendo encefalitis, trastornos cardiovasculares y hepatitis (3).

El CHIKV puede circular de manera endémica y causar brotes de rápida propagación; las áreas endémicas incluyen a Asia y África. En diciembre de 2013, la Organización Panamericana de la Salud emitió una alerta epidemiológica por presencia de casos autóctonos de chikungunya en la isla caribeña de Saint-Martin (4). En pocos meses, el virus se propagó al Caribe, la Florida en los Estados Unidos de América, América Central y América del Sur (5). El primer caso importado de chikungunya en Nicaragua fue registrado en julio de 2014 en el municipio de Somotillo, Chinandega (6). En setiembre del mismo año se identificó el primer caso autóctono de chikungunya en el país. A partir de esta fecha y hasta setiembre del 2015 se han reportado 6 694 casos confirmados y 64 097 casos sospechosos en Nicaragua (7, 8). Sin embargo, estos datos no son suficientes para estimar la incidencia real de infecciones por CHIKV, ya que la infección puede cursar de forma asintomática o puede causar síntomas leves que no inducen a las personas a realizar una consulta médica. El Ministerio de Salud de Nicaragua realizó un estudio en el mes de octubre del 2015 con el objetivo de determinar la seroprevalencia, la tasa de ataque clínica y la proporción de infecciones subclínicas en la población nicaragense mayor de dos años de edad.

## MATERIALES Y MÉTODOS

### Sujetos de estudios y estimación de la muestra

Se realizó un estudio transversal en 39 sitios, a nivel de los 19 Sistemas Locales de Atención Integral en Salud (SILAIS) del país, bajo los siguientes criterios: un municipio de alta incidencia y otro de baja incidencia (6, 7) por cada SILAIS (excepto el SILAIS Managua, donde se programaron dos distritos por ser la ciudad capital, donde se concentra una mayor cantidad de habitantes), con una prevalencia esperada de 10%, nivel de confianza de 95%, precisión de 5%, 2% por efecto de diseño y 10% de no respuesta. Estos cálculos se realizaron para cada uno de los SILAIS. El número de individuos a entrevistar fue de 10 712, para un tamaño de muestra definitivo de 11 792 personas. Por cada municipio o distrito seleccionado se eligió la zona a muestrear de forma aleatoria. El número de personas promedio sujetas a entrevistar en cada municipio o distrito fue de 302, para un promedio de 55 casas por municipio o distrito. La selección de las viviendas se realizó de manera aleatoria y en cada vivienda se ofreció la participación del estudio a todos los habitantes presentes al momento de la visita.

### Consideraciones éticas

El protocolo del estudio contó con la aprobación del Comité Institucional de Revisión Ética del Ministerio de Salud. Se solicitó consentimiento informado a los mayores de 16 años. En el caso de los menores de 16 años, se pidió consentimiento de los tutores y asentimiento verbal en los niños de 6 a 15 años.

### Recolección y procesamiento de la información

Un equipo de expertos del Ministerio de Salud, con el apoyo de la Organización Panamericana de la Salud y del Instituto de Ciencias Sostenibles, coordinaron y capacitaron a los 19 epidemiólogos de los SILAIS, a quienes se les explicó los objetivos, alcances del estudio, el manejo de muestras y del instrumento de recolección de datos. Luego, los epidemiólogos capacitaron y supervisaron a los equipos municipales responsables de la recolec-ción de la información y la toma de muestra. Se definió un algoritmo de toma, envío y recepción de muestras de los niveles participantes del estudio: municipio, SILAIS y Centro Nacional de Diagnóstico y Referencia (CNDR). Se separaron dos alícuotas: una para procesamiento y otra para almacenamiento, esta última en el caso de que el participante aceptara. El periodo de recolección de la información y toma de la muestra tuvo una duración de ocho días.

Se diseñó una base de datos en Microsoft Access versión 2013® para la introducción y procesamiento de información de las encuestas realizadas. Se crearon reglas de validación para las variables edad, SILAIS y municipio. Además de estos controles, las encuestas fueron doblemente digitadas con el fin de minimizar errores. Cada encuesta estuvo identificada por un código único consecutivo asignado a cada municipio. La digitación de la información se realizó en un periodo de diez días por un equipo de diez digitadores. Se utilizó el programa EpiInfo versión 3.0® para comparar la doble digitación y el programa estadístico STATA versión 13® para el análisis de los datos.

### Procedimientos de muestras serológicas

Las muestras fueron procesadas por el método ELISA de inhibición para detectar anticuerpos anti-CHIKV, desarrollados por el CNDR (Balmaseda, co-municación personal). Se trata de una técnica competitiva donde los anticuerpos específicos contra el CHIKV presentes en las muestras de sueros de los participantes compiten con un conjugado (anticuerpo unido a una enzima) anti-CHIKV por el antígeno viral. Se consideraron positivas las muestras de suero mayor o igual del 50% de inhibición en una dilución 1:10.

### Definiciones y análisis estadístico

La seroprevalencia se definió como el número total de positivos por MEI entre el total de participantes con muestras serológicas. La tasa de ataque clínica fue definida como el total de personas que respondieron que se habían enfermado por chikungunya y, además, tuvieron confirmación por laboratorio entre el total de participantes con muestras serológicas. La proporción de infecciones subclínicas se calculó como el porcentaje de personas que respondieron no haberse enfermado y que resultaron positivos por MEI. El índice de vivienda es el porcentaje de viviendas positivas para larvas de *Aedes aegypti* con respecto al total de viviendas inspeccionadas en una localidad y se calculó de forma mensual. Para resumir los datos, se mostraron frecuencias absolutas y relativas para datos categóricos. En cuanto a las variables edad y personas por casas, se calcularon la media y la desviación estándar. Se utilizaron modelos lineales generalizados y modelos de multinivel de Poisson para estimar las razones de prevalencia crudas y ajustadas respectivamente, con sus respectivos intervalos de confianzas de 95%.

## RESULTADOS

### Datos generales de los participantes

Se enrolaron un total 11 722 participantes y se obtuvieron 11 449 (97,6%) muestras serológicas. Del total de muestras, 11 280 (98,5%) fueron procesadas y 169 (1,5%) no se pudieron procesar por tener un volumen insuficiente (cuadro 1). La distribución de los participantes y muestras tomadas fue similar en los 19 SILAIS, con aproximadamente 550 a 600 participantes por cada uno de ellos repartidos en dos municipios, con la excepción del SILAIS Managua, donde se seleccionaron tres localidades (dos distritos y un municipio), donde, por lo tanto, se enroló el mayor número de participantes. Se observó un predominio del sexo femenino (67,8%) y la edad promedio fue de 34 años con una desviación estándar (DE) de 19,5. El promedio de habitantes en las viviendas encuestadas fue 5,2 (DE: 2,6) (cuadro 1).

**CUADRO 1.** Distribución por SILAIS del sexo, edad promedio, promedio de habitantes por casas y muestras serológicas, Nicaragua, 2015

SILAIS	Participantes	Sexo femenino		Edad promedio		Habitantes por casa		Muestras serológicas
		N	%	Promedio	DE	Promedio	DE	
Bilwi	600	366	61,0	28	17,6	5,8	2,9	593
Boaco	600	418	69,7	36	21,3	4,1	1,9	571
Carazo	615	428	68,6	39	19,6	5,2	2,7	565
Chinandega	607	395	65,1	34	18,8	5,1	2,1	566
Chontales	613	435	71,0	31	18,5	5,0	2,0	605
Esteli	650	442	68,0	38	21,3	4,8	2,2	587
Granada	599	427	71,3	35	19,7	6,1	2,8	586
Jintega	591	431	72,9	36	18,2	5,2	2,6	579
León	619	418	67,5	36	20,1	4,8	2,3	580
Madriz	564	349	61,9	35	20,0	4,9	1,9	548
Managua	907	623	68,0	35	19,1	5,8	2,6	885
Masaya	561	364	64,9	35	20,5	5,9	2,6	552
Matagalpa	565	405	71,7	34	19,9	5,5	2,6	562
Las Minas	608	402	66,1	31	17,8	5,2	2,8	590
Nueva Segovia	608	394	65,7	31	17,9	5,1	2,0	594
RACCS	633	433	68,4	31	18,3	4,9	2,3	602
Río San Juan	606	413	68,2	28	17,9	5,3	2,2	588
Rivas	576	372	64,6	37	18,4	4,3	2,3	548
Zelaya	608	436	71,7	31	21,1	4,9	2,1	579
Total país	11 722	7 951	67,8	34	19,5	5,2	2,4	11 280

SILAIS, Sistemas Locales de Atención Integral en Salud; RACCS, Región Autónoma de la Costa Caribe Sur; DE, desviación estándar.

### Seroprevalencia, tasa de ataque clínica y proporción de infecciones subclínicas en el nivel nacional

Se analizaron los datos para calcular la seroprevalencia, la tasa de ataque clínica y la proporción de infecciones subclínicas por virus chikungunya en el nivel nacional (8). Se observó una seroprevalencia de 32,8% (IC95%: 31,9-33,6), una tasa de ataque clínica de 26,5% (IC95%: 25,7-27,3) y una proporción de infecciones subclínicas de 19,1% (IC95%: 17,8-20,4) (cuadro 2). Se observaron diferencias significativas de seroprevalencia entre los sexos, con un predominio del sexo femenino de 3,6 puntos porcentuales (IC95%: 1,8-5,5). En cuanto a los grupos etarios, existe una tendencia al incremento hasta el rango de 40-59 años, que disminuye en el rango de 60 años y más. No se observaron diferencias significativas en la seroprevalencia según el número de personas por casa (cuadro 2).

Para el cálculo de la tasa de ataque clínica, se consideraron como casos clínicos aquellos participantes que reportaron haber presentado cuadro clínico de fiebre chikungunya y que fueron confirmados MEI. Se observaron diferencias significativas entre los sexos siendo mayor la tasa de ataque en el sexo femenino con 5,1 puntos porcentuales más altos (IC95%: 3,4-6,8). En cuanto a los grupos etarios y el número de personas por casa, el comportamiento fue similar al de seroprevalencia (cuadro 2).

La proporción de infecciones subclínicas fue mayor en el sexo masculino, con una diferencia de 6,9 puntos porcentuales (IC95%: 3,9-9,8). En cuanto a los grupos etarios, se observó que fue relativamente más alta en los rangos de 2-4 años y de 60 años o más (cuadro 2).

**CUADRO 2.** Seroprevalencia, tasa de ataque clínica y proporción de infecciones subclínicas por chikungunya, Nicaragua, 2015

Variables	Seroprevalencia		Tasa de ataque subclínica		Proporción de infecciones subclínicas	
	%	IC95%	%	IC95%	%	IC95%
Sexo						
Masculino	30,3	28,8-31,8^a^	23,1	21,7-24,5	23,9	21,4-26,5
Femenino	33,9	32,9-35,0^a^	28,2	27,1-29,^a^	17,0	15,6-18,5^a^
Edad (años)						
2 a 4	21,3	16,7-26,4	16,0	11,9-20,8	24,6	14,5-37,3
5 a 10	24,2	21,2-27,4	19,2	16,5-22,2	20,5	15,0-26,9
11 a 15	31,0	28,0-34,2	24,7	21,9-27,6	20,4	15,6-25,6
16 a 39	34,0	32,8-35,3	27,9	26,7-29,1	17,9	16,3-19,8
40 a 59	35,0	33,1-36,9	28,9	27,1-30,7	17,4	14,9-20,0
60 y más	32,2	29,7-34,7	24,3	22,0-26,6	24,7	20,8-28,9
Habitantes por casa						
1 a 4	33,7	32,4-35,0	26,9	25,7-28,2	20,0	18,1-21,9
5 y más	32,0	30,9-33,2	26,2	25,1-27,3	18,3	16,6-20,1

a Diferencias significativas a un nivel de 5%. IC95%, inter valo de confianza de 95%.

### Análisis multivariado y multinivel de la seroprevalencia

Las razones de seroprevalencia por sexo, grupo etario y número de personas por casa fueron estimadas mediante un modelo lineal generalizado. Además, las razones de seroprevalencia se ajustaron por localidad en un modelo multinivel de Poisson. De acuerdo al modelo ajustado, no hay diferencia significativa en la seroprevalencia por sexo (cuadro 3). Con respecto a la edad, se observó que la seroprevalencia más baja fue en los niños de 2-4 años (cuadro 3). Además, el grupo de 5-10 años también presentó una seroprevalencia menor que en los grupos de mayor edad (razón de prevalencia del grupo de 11-15 años en comparación con el de 5-10 años: 1,26, IC95%: 1,05-1,52). Por último, la seroprevalencia en los participantes que viven en casas de cinco o más habitantes fue menor que en las casas con uno a cuatro habitantes, aunque el efecto en este indicador fue mínimo (cuadro 3).

**CUADRO 3.** Modelo de análisis multinivel de la razón de prevalencia del sexo ajustado por grupos etarios y habitantes por casa, Nicaragua, 2015

Variables	Razón deprevalencia cruda	IC95%	Razón prevalencia ajustada	IC95%
Sexo				
Masculino	1	NA	1	NA
Femenino	1,11	1,05-1,18%^a^	1,07	0,99-1,15
Edad (años)				
2 a 4	1	NA	1	NA
5 a 10	1,13	0,89-1,45	1,36	1,02-1,82%^a^
11 a 15	1,46	1,15-1,84%^a^	1,72	1,30-2,27%^a^
16 a 39	1,60	1,28-1,99%^a^	1,79	1,38-2,31%^a^
40 a 59	1,64	1,31-2,05%^a^	1,82	1,40-2,37%^a^
60 y más	1,51	1,20-1,90%^a^	1,67	1,28-2,19%^a^
Habitantes por casa				
1 a 4	1	NA	1	NA
5 y más	0,95	0,90-1,01	0,92	0,86-0,98%^a^

a Diferencias significativas a un nivel de 5%. IC95%, inter valo de confianza de 95%.

### Seroprevalencia de chikungunya en los sitios del estudio

**FIGURA 1.**
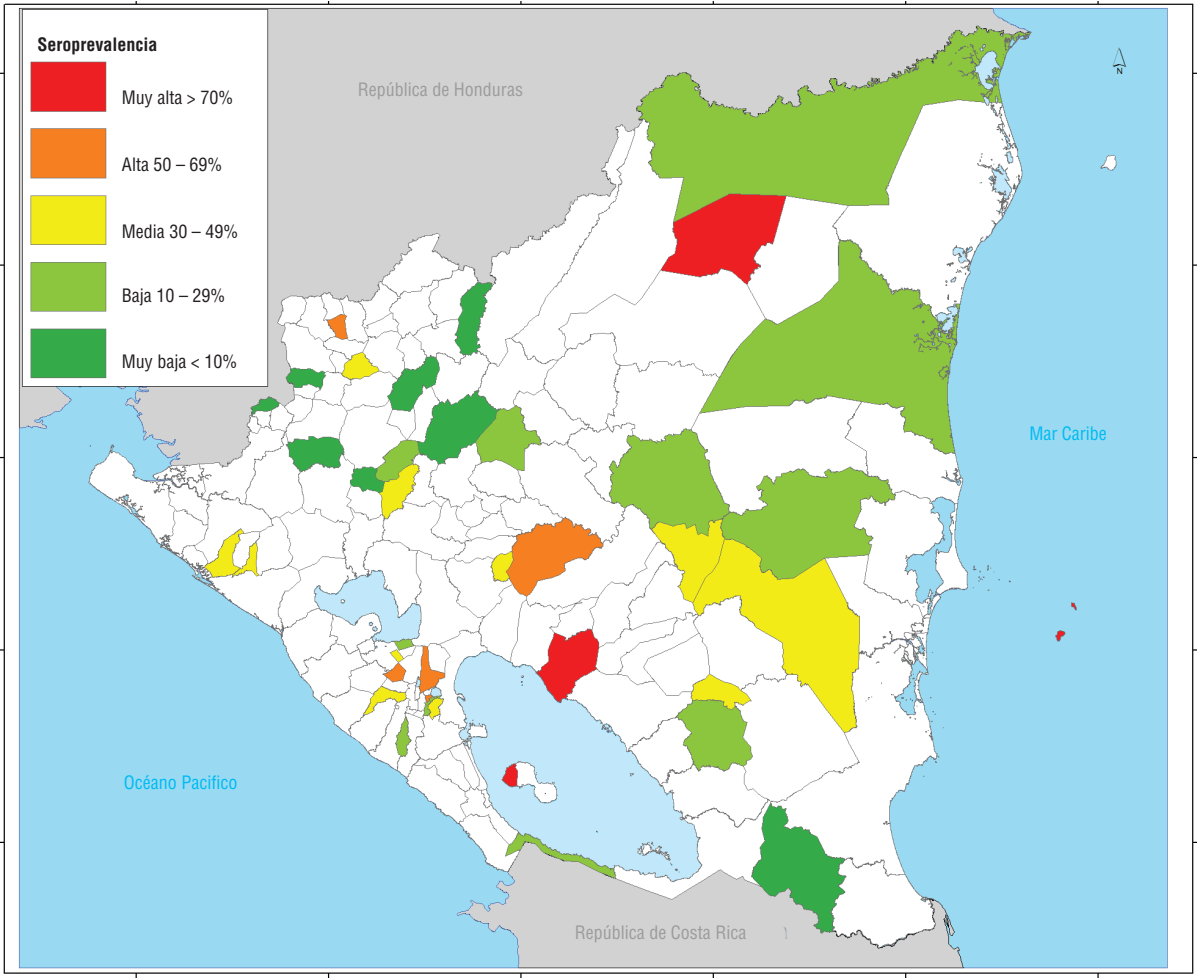
Distribución de la seroprevalencia de virus chikungunya a nivel de SILAIS y municipio, Nicaragua, 2015.

**FIGURA 2.**
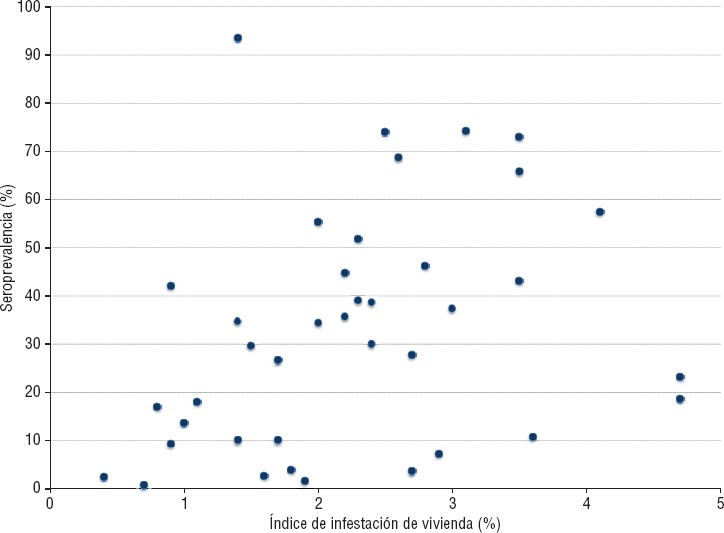
Diagrama de dispersión entre seroprevalencia de chikungunya e índice de infestación de vivienda por el mosquito *Aedes aegypti*, Nicaragua, 2015.

La seroprevalencia de chikungunya fue calculada en cada una de los 39 sitios seleccionados para el estudio. Se observaron grandes diferencias en la seroprevalencia por localidad, con un rango de 0,7% (IC95%: 0,08-2,5) en Yalí, en el SILAIS Jinotega, a 73% (IC95%: 67,7 - 77,9) en Juigalpa, en el SILAIS Chontales (figura 1). Se analizó la relación entre la seroprevalencia a nivel de la localidad y el promedio de los índices mensuales de infestación de vivienda por *Aedes aegypti* en el período de setiembre de 2014 a setiembre de 2015. En la figura 2 se observa que la tasa de seroprevalencia a nivel de la localidad aumenta con el índice de infestación de la vivienda. La razón de seroprevalencia cruda por unidad de índice de infestación fue de 1,23 (IC95%: 0,99-1,52). Posteriormente, se construyó un modelo multivariado incluyendo la densidad poblacional de la localidad así como su altura. En este modelo, solo el índice de infestación resultó significativo con una razón de seroprevalencia ajustada de 1,82 (IC95%: 1,17-2,84).

## DISCUSIÓN

Desde la alerta epidemiológica de diciembre del 2013 emitida por la Organización Panamericana de la Salud/Organización Mundial de la Salud (OPS/OMS), la propagación de la enfermedad en la Región de las Américas hasta noviembre del 2015 alcanzó 33 países. Durante el año 2014, la notificación de casos de chikungunya en el nivel subregional en Nicaragua representó menos de 1%; sin embargo, para el año 2015 se notificaron 30,7% de los casos. Durante el año 2015 se reportaron casos en todo el territorio nacional con incrementos por pico estacional y brotes de gran magnitud (4, 5).

Considerando las fortalezas del Modelo de Salud Familiar y Comunitario (MOSAFC) con base en la participación comunitaria y el acceso a los servicios de salud (9), y tomando en cuenta los esfuerzos desarrollados en la vigilancia de fase preparatoria y respuesta (10) implementados desde marzo del año 2014, el Ministerio de Salud de Nicaragua requería estimar la seroprevalencia y la tasa de ataque clínica de la enfermedad para reorientar las medidas pertinentes en la gestión integrada de la prevención y control de la enfermedad mediante el refuerzo de componentes de vigilancia, atención al manejo de casos, comunicación social y participación comunitaria e intersectorial.

Este estudio es el primer reporte de seroprevalencia desde la introducción del virus del chikungunya en el continente americano en diciembre de 2013 (4, 11). En él se reporta la seroprevalencia de infecciones por el virus de chikungunya en Nicaragua después de su introducción entre agosto y de 2014. El estudio se realizó en 39 sitios del país (37 municipios y dos distritos de Managua, la capital) y se enrolaron en total 11 722 participantes de dos años o más. En el nivel nacional, se registró una seroprevalencia de 33% y una tasa de ataque clínica de 26,5%. Además, se observó que la proporción de infecciones subclínicas fue de 19,1%.

Se han realizado varios estudios de seroprevalencia de chikungunya en todo el mundo. Estos estudios describen seroprevalencias de 10% en el noreste de Italia (12), 37% en Mayotte (13), 38% en Reunión (14), 56% en Bagan Panchor, Malasia (15), 63% en las Comoras (16), 68% en Kerala, India (17), y 75% en la isla de Lamu, Kenia (18). Sin embargo, resulta difícil comparar los resultados obtenidos en Nicaragua con otros estudios, ya que estos se realizaron en situaciones geográficas diferentes, algunos en contexto de brotes (12–14, 16) y otros en zonas endémicas (15, 17, 18). Además, algunos de estos estudios corresponden a genotipos virales diferentes (12–17) del genotipo asiático que ha estado circulando en la región (11, 19). Finalmente, otros factores como el nivel de inmunidad poblacional, factores genéticos, los vectores causales y las medidas de control vectorial también podrían tener un impacto sobre la seroprevalencia.

Una comparación más certera podría ser con la situación en República Dominicana, país que se ha visto afectado por el chikungunya desde la introducción del virus en la Región. La tasa de ataque clínica en Nicaragua reportada en este estudio, de 26,5%, es inferior en comparación con las tasas de ataque de República Dominicana que van de 66% a 87% (20, 21). Esto puede deberse a factorescomo la densidad poblacional; sin embargo, es posible que el funcionamiento exitoso del modelo de salud donde hay intervenciones a gran escala en el control vectorial y una activa participación comunitaria hayan incidido de manera sustancial en la disminución de la tasa de ataque. El reforzamiento de la vigilancia en los puntos de entrada que retrasó desde julio a septiembre del 2014 la transmi-sión autóctona también pudo haber influido en la disminución de la tasa de ataque clínica.

A nivel individual, se observó que la seroprevalencia de chikungunya es similar en mujeres y hombres. Otros estudios de seroprevalencia a través del mundo han mostrado datos variables con respecto a la seroprevalencia de chikungunya: se observó predominio masculino (12, 13, 17), predominio femenino (15, 16) o valores similares en ambos sexos (18). En nuestro estudio, la seroprevalencia más baja se observó en los niños de 2 a 4 años, seguidos por los de 5 a 10 años. Los participantes de 11 años y más presentaron la seroprevalencia más alta. La mayoría de los otros estudios que analizaron la seroprevalencia por edad muestran la misma tendencia: una menor seroprevalencia en los niños (12, 13, 15, 17, 18). Esto se podría explicar porque los adultos pasan más tiempo fuera de la casa, en lugares donde tienen un riesgo más elevado de infección que el domicilio, donde las familias hacen más énfasis en la práctica de medidas preventivas (por ejemplo, uso de repelente y mosquiteros).

La seroprevalencia en los 39 sitios seleccionados varió de forma significativa, de menos de 1% a más de 70% y, a menudo, municipios cercanos presentaron seroprevalencias muy diferentes. Esta gran heterogeneidad geográfica se ha observado en otros países (13, 22). Además, se correlaciona la seroprevalencia de la localidad con algunos factores ambientales y demográficos. En particular, se observó que la seroprevalencia es mayor en los sitios con mayores tasas de infestación por el mosquito *Aedes aegypti*. Una correlación similar se observó en un estudio en la India (23), donde existió un incremento de la seroprevalencia de chikungunya con el aumento de los índices entomológicos de mosquitos *Aedes*.

La proporción de infecciones subclínicas de 19,1 % en el presente estudio está dentro de los rangos de 17,5 a 27,8% registrados en otros estudios (12, 13). Es notable que la proporción de infecciones subclínicas fue mayor en los hombres (23,9%) que en las mujeres (17%). Por lo contrario, no se observaron diferencias significativas en la proporción de infecciones subclínicas por edad.

Una limitación de este estudio fue que la tasa de ataque clínica y la proporción de infecciones subclínicas se estimaron según la percepción del participante. Sin embargo, 78,7% (IC95%: 77,3-80,0) de los que manifestaron haber tenido la enfermedad tuvieron la confirmación por laboratorio, lo que demuestra asociación entre la percepción de la persona y la confirmación serológica de la infección por CHIKV. Aun así, estos indicadores podrían estar afectados por el sesgo de recuerdo y por el conocimiento de los participantes sobre esta enfermedad. Otra limitación es que la gran variación geográfica observada en las tasas de seroprevalencia en los diferentes sitios del estudio puede limitar la capacidad de inferir la tasa real de seroprevalencia en el resto del país. La utilización de una técnica de ELISA podría ser considerada como una limitación de ese estudio debido a la posibilidad de reacciones cruzada con otros alfavirus. Sin embargo, la técnica fue optimizada con muestras tomadas antes de la introducción del CHIKV en Nicaragua, mostrando una excelente especificidad (24).

En conclusión, este estudio constituye el primer reporte de seroprevalencia de infección por chikungunya en la Región, por lo que aporta información invaluable sobre la introducción y la dinámica de transmisión de esta enfermedad en Nicaragua. El modelo utilizado, con una confirmación de las infecciones utilizando una técnica casera validada, una amplia participación comunitaria y el rol rector del Ministerio de Salud en el nivel local, departamental y nacional, constituye un referente para la realización de estudios similares en la región.

## Agradecimientos

Los autores desean agradecer a la población de Nicaragua, que permitió alcanzar el objetivo del estudio al haber obtenido una gran aceptación en la toma de muestra y en el llenado de la encuesta; a los equipos de trabajo de los Sistemas Locales de Atención Integral de la Salud y municipales, quienes coordinaron y ejecutaron con éxito la recolección de la información; a la Organización Panamericana de Salud y al Instituto de Ciencias Sostenibles, por su cooperación técnica y financiera, y a Eva Harris por su contribución en el estudio.

## Declaración

Las opiniones expresadas en este manuscrito son responsabilidad del autor y no reflejan necesariamente los criterios ni la política de la *RPSP/PAJPH* y/o de la OPS.
